# KLSD: a kinase database focused on ligand similarity and diversity

**DOI:** 10.3389/fphar.2024.1400136

**Published:** 2024-06-18

**Authors:** Yuqian Yuan, Xiaozhu Tang, Hongyan Li, Xufeng Lang, Can Li, Yihua Song, Shanliang Sun, Ye Yang, Zuojian Zhou

**Affiliations:** ^1^ School of Artificial Intelligence and Information Technology, Nanjing University of Chinese Medicine, Nanjing, China; ^2^ School of Medicine and Holistic Integrative Medicine, Nanjing University of Chinese Medicine, Nanjing, China; ^3^ National and Local Collaborative Engineering Center of Chinese Medicinal Resources Industrialization and Formulae Innovative Medicine, Jiangsu Collaborative Innovation Center of Chinese Medicinal Resources Industrialization, Jiangsu Key Laboratory for High Technology Research of TCM Formulae, Nanjing University of Chinese Medicine, Nanjing, China

**Keywords:** kinase, database, SMKIs, pharmacological similarity, ligand activity

## Abstract

Due to the similarity and diversity among kinases, small molecule kinase inhibitors (SMKIs) often display multi-target effects or selectivity, which have a strong correlation with the efficacy and safety of these inhibitors. However, due to the limited number of well-known popular databases and their restricted data mining capabilities, along with the significant scarcity of databases focusing on the pharmacological similarity and diversity of SMIKIs, researchers find it challenging to quickly access relevant information. The KLIFS database is representative of specialized application databases in the field, focusing on kinase structure and co-crystallised kinase-ligand interactions, whereas the KLSD database in this paper emphasizes the analysis of SMKIs among all reported kinase targets. To solve the current problem of the lack of professional application databases in kinase research and to provide centralized, standardized, reliable and efficient data resources for kinase researchers, this paper proposes a research program based on the ChEMBL database. It focuses on kinase ligands activities comparisons. This scheme extracts kinase data and standardizes and normalizes them, then performs kinase target difference analysis to achieve kinase activity threshold judgement. It then constructs a specialized and personalized kinase database platform, adopts the front-end and back-end separation technology of SpringBoot architecture, constructs an extensible WEB application, handles the storage, retrieval and analysis of the data, ultimately realizing data visualization and interaction. This study aims to develop a kinase database platform to collect, organize, and provide standardized data related to kinases. By offering essential resources and tools, it supports kinase research and drug development, thereby advancing scientific research and innovation in kinase-related fields. It is freely accessible at: http://ai.njucm.edu.cn:8080.

## Graphical Abstract

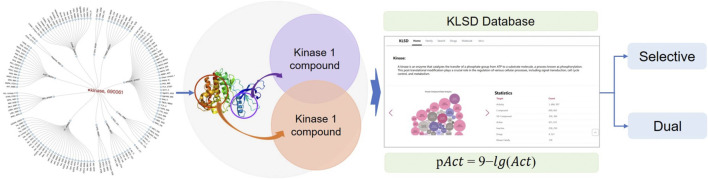

## 1 Introduction

Protein kinase is an important signal messenger that plays a crucial role in cellular activities. It functions as an enzyme that specifically transfers the γ-phosphate group from ATP to amino acid residues on substrate proteins. This phosphorylation enables the transmission of various signals that are necessary for numerous physiological processes (Fischer, 2016). Pharmacological and pathological studies have shown that kinases are an ideal and important drug targets for drug development in the treatment of diseases such as tumors, inflammatory diseases, central nervous system diseases, cardiovascular diseases and diabetes (Wu et al., 2015). Kinases typically contain an active center that plays a key role in catalyzing phosphorylation reactions. These enzymes are often regulated through the binding of other proteins, phosphorylation events, and the influence of other molecules. Given the significant biological functions of kinases in organisms and their potential as drug targets (Mukhtar et al., 2017), many researchers have been focused on studying SMKIs. These SMKIs can be used to modulated aberrant signaling pathways and treat a variety of diseases as mentioned. As science and technology advance, the proliferation of biomedical databases continues (Attwood et al., 2021; Cohen et al., 2021). However, language barriers, heavy workloads, and other obstacles often limit us to a few well-known databases. Heavy workloads often involve tasks such as conducting kinase target difference analysis, which may require importing kinase data into EXCEL, CSV, etc., for manual calculation, screening standard type and relationship manually, and calculating standard values. These tasks can significantly increase the workload of the study. Additionally, other obstacles, such as a cluttered website layout that makes it difficult to quickly locate research-related content, can also present challenges. This widespread reliance on popular databases restricts novel discovery opportunities and hinders innovation. In particular, research involving small molecule kinase inhibitors (SMKIs) critically requires a comprehensive pharmacological database. A kinase database that maintains strict integrity, provides high-quality data, and offers extensive customization is invaluable. It is imperative to venture beyond familiar grounds and delve into specialized, lesser-known research databases to discover and utilize new data resources, driving pioneering breakthroughs.

With the continuous development of artificial intelligence (AI) technology, significant progress has been made in kinase informatics research. In drug research, AI technology can leverage various molecular representation properties, such as molecular graph-based feature representation (Duvenaud et al., 2015; Kevin et al., 2019; Mengying et al., 2020), molecular string-based representation (Nathan et al., 2018; Popova et al., 2018; Karpov et al., 2020) image-based representation (Charles et al., 2018; Cortés-Ciriano and Bender, 2019), and knowledge-based molecular representation (Jan et al., 2019; Xiang et al., 2021). This allows for satisfactory performance in the research and analysis of various drug properties, including activity, pharmacokinetic properties, and toxicological properties. This development is conducive to facilitating early drug discovery. The advantages of AI technology in kinase informatics research are mainly reflected in the following aspects.

i) Rich data resources: Kinase data consolidates a vast amount of information about genes, protein structures, phosphorylation sites, regulatory pathways. This provides researchers with abundant data resources to explore the functions and roles of kinases.
ii) Diverse functional annotations: Kinase data not only offers basic information about kinases, but also encompasses functional annotations relating to cell signaling, disease development, and drug discovery. This comprehensive data can aid in a more thorough understanding of the biological roles of kinases.
iii) Bioinformatics tools: Numerous biological databases supply bioinformatics tools for data analysis, structure prediction, and protein interactions. These tools enable researchers to interpret and utilize data more effectively.
iv) Data integration and cross-linking: Large-scale biological databases integra data from various sources, providing cross-linkages to help researchers conduct more in-depth studies using integrated data.

Although kinase informatics research has achieved significant breakthroughs, there are still deficiencies in data quality consistency, data standardization, data visualization, personalized functional analysis, and prediction. Currently, there is a lack of specialized databases for kinases in the field of kinase informatics research. The KLIFS (Kanev et al., 2021) (Kinase-Ligand Interaction Fingerprints and Structures) database is one such specialized database. Obviously, there are differences in focus and application areas between the KLIFS database and the KLSD database discussed in this paper. KLIFS primarily focuses on kinase structures and co-crystalized kinase-ligand interactions, facilitating kinase drug discovery and design. On the other hand, KLSD emphasizes all reported SMKIs analysis between kinase targets. Therefore, based on the current foundations of kinase data, further research is being conducted to construct a professional and personalized kinase database. This database not only provides comprehensive data on the structure, function, regulation and biological roles of kinase-related proteins, but also helps to understand interactions between kinases and other proteins, molecules, or biological systems through the analysis of target difference analysis. This comprehensive understanding of kinase systems better support both scientific research and design, as well as signaling applications.

## 2 Materials and methods

### 2.1 Data collection and processing

**FIGURE 1:**
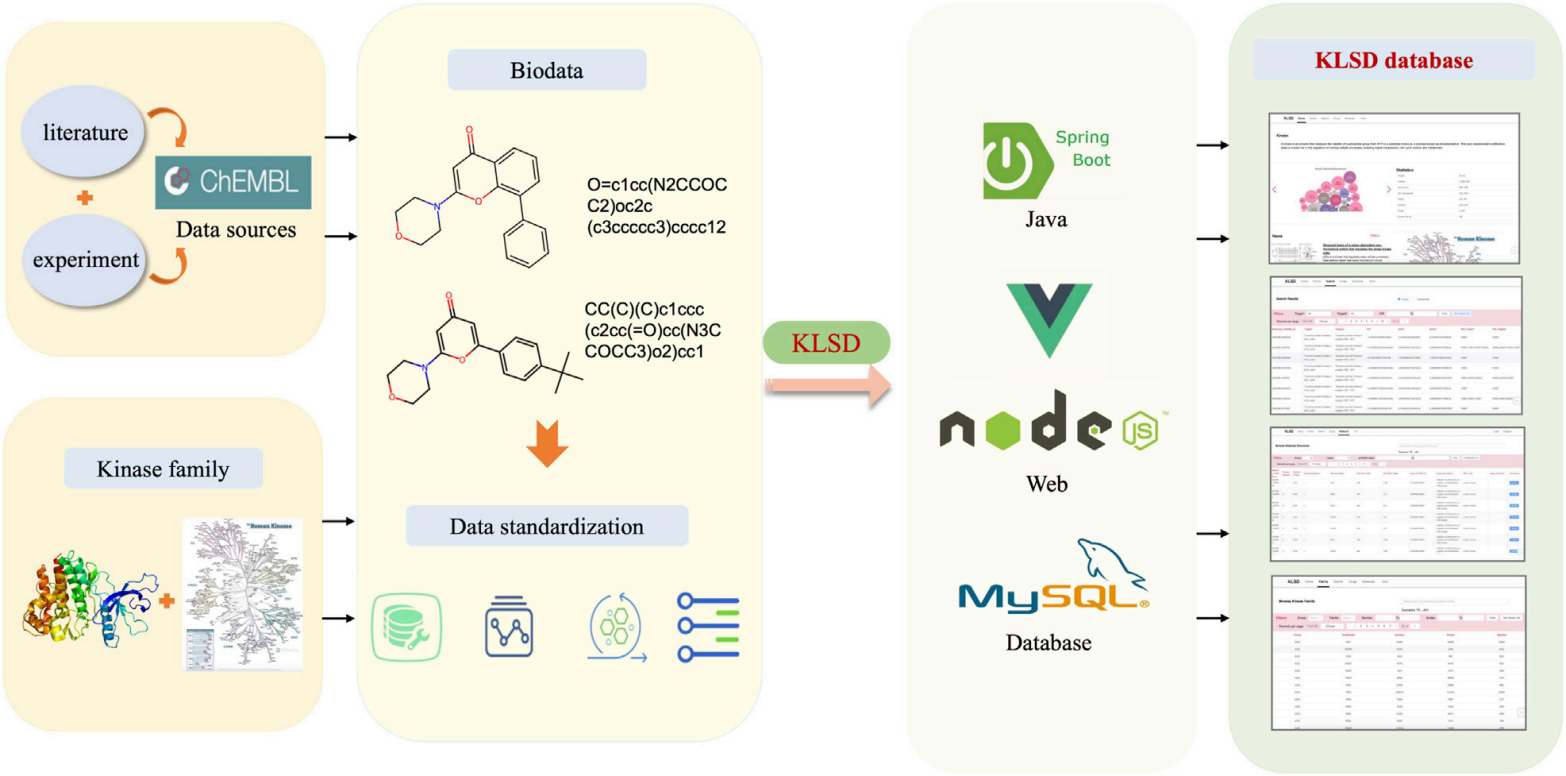
Comprehensive Architecture of the KLSD Database Platform. The KLSD database emphasizes the analysis of SMKIs among all reported kinase targets.

The KLSD database platform is mainly based on the data in the ChEMBL (Chemical Database of Bioactive Molecules) database (Mendez et al., 2019). The data in the ChEMBL database is primarily derived from scientific literature, patent information, public databases, high-throughput screening data, clinical trials and drug registration information. These multiple data sources and channels ensure the accuracy and comprehensiveness of the data. Therefore, for our experiments, we used data from the CHEMBL32 version of the ChEMBL database. ChEMBL currently provides multiple publicly available web services for accessing compound and bioactivity data in their database. Through their web service, we obtained a total of 78 data tables with 57,424,581 records. These tables cover various of aspects such as genomics, protein expression, small molecule, system, ontologies and scientific literature. with 15,398 kinds of targets of all kinds and more than 2,390,000 different compounds. Since the focus of this study is mainly related to kinases, we selectively extract kinase-related data to build a kinase dataset. By conducting a keyword search, we initially obtained 1,131,037 kinase-related records. The data extraction process and platform construction is shown in Figure 1.

In biology, hierarchical classification aids in better understanding the similarities and differences among kinases, as well as their roles in cell signaling and biological processes. Thus, we employed the classification framework provided by Manning et al. (Manning et al., 2002), which is based on sequence homology and functional characteristics to categorize kinases into groups, families, and subfamilies. Kinases are first grouped based on their overall structural domains and sequence similarities of their kinase domains. Within each group, kinases are further classified into families using more specific sequence similarities and shared functional characteristics. Subsequently, within each family, subfamilies are established, which may consist of different forms of a single gene product or closely related genes with similar functions. This classification method is commonly used in biological research and drug development, aiding in the identification of relationships among different kinases and improving our understanding of their intricate roles in various signaling pathways and diseases. This process resulted in a total of 14 clusters, 166 families, and 314 subfamilies.

**FIGURE 2:**
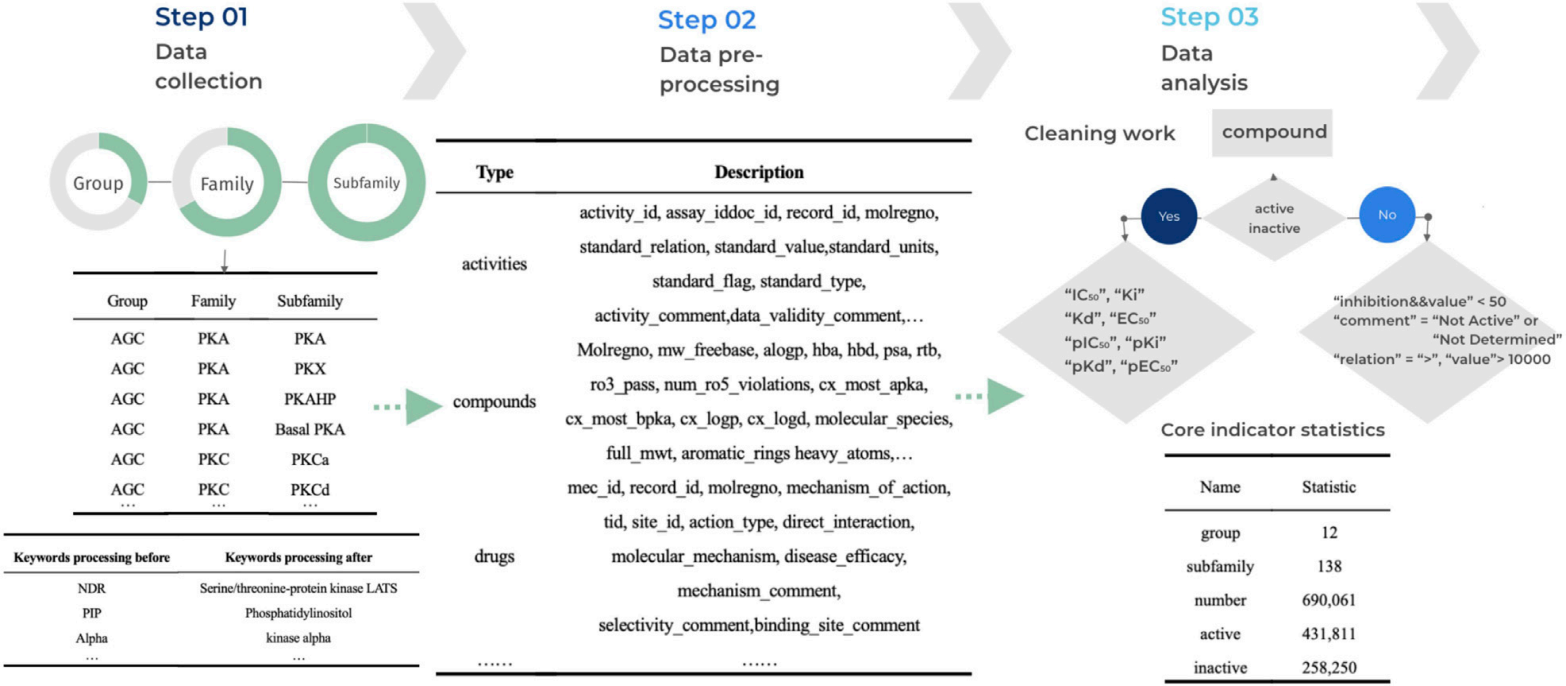
Workflow for KLSD Data Processing and Standardization. It describles the key aspects of data processing—including cleaning, standardization, and normalization.

Data processing refers to the process of performing a series of operations and transformations on raw data to obtain valuable information. These operations can include extracting specific features, cleaning the data, eliminating noise, and converting the formats, among others (Han et al., 2022). The purpose of data processing is to make the data more useable, comprehensible, and applicable for further analyses, applications, and decision-making. To effectively improve the data quality of kinase data, reduce errors and bias in the analysis, and facilitate later data analysis, mining and modelling for more accurate and meaningful conclusions, we have implemented standardization measures for the data. This ensures data consistency, comparability and accuracy. The initial step involves processing data outlier, where we eliminate any potential outliers, such as invalid entries and missing values, to maintain data integrity. We consider an outlier when the data value is null or empty, or when the data value has an abnormal symbol such as at, #, etc. If the length of the data value falls outside the normal range of values for that property, we consider that data is invalid. Following this, we standardize the data by aligning it across different sources, including the Standard Type of compounds and Standard Relation. This standardization enables consistent querying and analysis within the database. The final step is data normalization, where we establish rules to standardize common abbreviations or variants by replacing them with their canonical nomenclature. For instance, the abbreviation ‘pip’ is replaced with ‘phosphatidylinositol’ to maintain uniformity throughout the data. The detailed process of kinase data processing is illustrated in Figure 2.

When working with kinase data, it is important to note that our focus should be on kinase target information. Targets typically refer to molecules, proteins, or other biomolecules that are either the object of a drug’s action or a key component of a signaling pathway. These targets are crucial in drug discovery as they serve as possible sites of drug action and are the protein molecules that interact with a drug to produce a specific biological effect. Target data consists of information such as substrate name, identifier, phosphorylation site information, phosphorylation specificity information, phosphorylation effect, substrate regulatory mechanism and biological function. The target site information of kinases plays a crucial role in understanding kinase function, cell signaling, and disease mechanisms. In this paper, we have meticulously documented and performed differential data analyses on kinase target information as part of our efforts to construct a kinase database platform. This paper aims to support scientific research and drug development related to kinase.

### 2.2 Database design and construction

**FIGURE 3:**
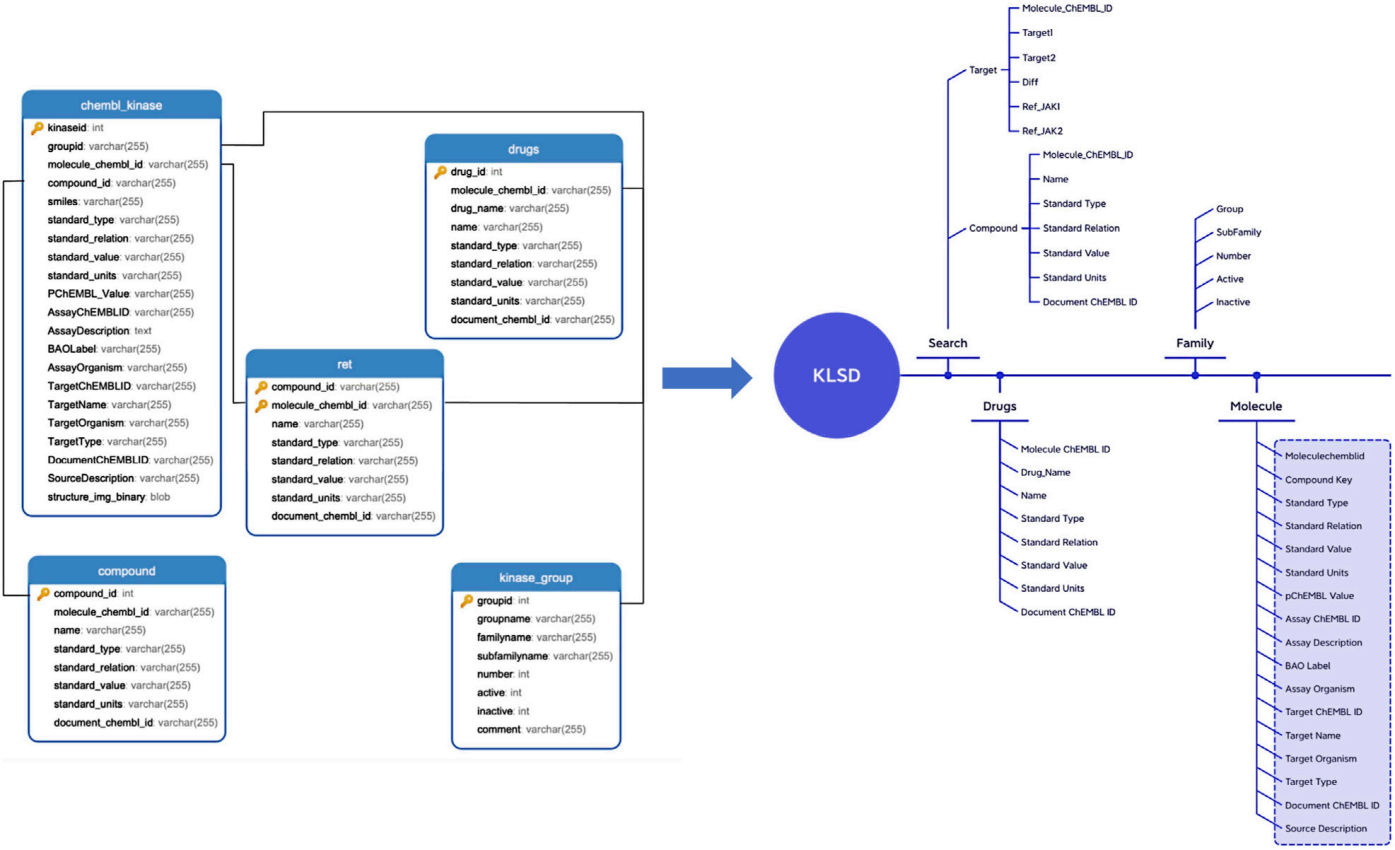
Detailed Data Mapping of the KLSD Database Platform. It describes the detailed mapping and the complex interconnections among various data types within the KLSD database platform.

KLSD platform serve as an open database for chemical biology and drug discovery, containing extensive compound and bioactivity data. It encompasses various data tables for the storage of different types of data. Table 1 illustrates some of the common data tables in the database and their key functions, such as assays, activities, compounds, targets, etc. Apart from these prevalent tables, KLSD also includes additional tables to accommodate a wider range of data types. Additional tables, such as the article table exists to contain kinase-related articles for researchers to keep up with progress on kinases, have been added accordingly in the text. The primary objective of KLSD is to provide researchers with open access information resources in the field of chemical biology and drug discovery, aiding their comprehension of the biological activities of kinase compounds. Special emphasis is placed on highlighting target information and target difference data of kinases as depicted in Figure 3.

**TABLE 1:** List and functions of data tables in the KLSD database.

Number	Name	Description
1	assays	Stores information about a bioassay experiment, including the type of experiment, objectives, literature citations, assay results, etc. The records in this table correspond to a bioassay experiment
2	activities	Contains bioactivity data such as bioactivity values for compounds, units, assay conditions, etc. This table is associated with the assays table, which correlates the results of bioassay experiments to compounds and targets
3	compounds	Stores information about the compound, including structure, chemical properties, identifiers, etc. Records in this table correspond to compound entities
4	targets	Contains information about biological targets, such as protein identifiers, names, classes, etc. This table is used to describe targets in drug development
5	docs	Store document information related to bioassay experiments and literature citations, such as literature titles, authors, abstracts, etc.
6	compound_structures	Contains structural information about the compound, such as a description of the two- or three-dimensional structure
7	bioactivities	This is a combined table containing activity data that brings together compounds, bioassay experiments and biological activity data
8	cell_lines	Store information about the cell line, including the source, type, and characteristics of the cell line. This is important for drug screening and bioassay experiments
9	tissue	Contains information about the tissue sample, such as tissue source, tissue type, etc. This is also important for describing the tissue expression of the target.
10	drug_indication	Used to store information about the indication for a drug (the disease or condition that the drug treats)
11	drug_mechanism	Contains mechanistic information about the drug, describing how the drug affects the biological target.

In this paper, we explore data representation and storage for kinase compounds’ molecular structures within the KLSD database. Compounds are primarily represented using SMILES (Simplified Molecular Input Line Entry System) strings and depicted through chemical molecular structure diagrams (Weininger, 1988). In kinase research, converting SMILES to chemical molecular structure diagrams is vital for understanding the topology, stereo configuration, and functional group positions of kinase molecules. This transformation aids in structure-activity relationship (SAR) analyses, which are critical for identifying structural features that influence kinase inhibitory activity. Thus, effectively translating SMILES into diagrams enhances drug design, virtual screening, SAR analysis, and the study of kinase-related diseases, playing a key role in kinase research and drug discovery (Li and Fourches, 2021).

In this paper, RDKit (Landrum, 2013) is mainly utilized for converting compound’s SMILES into molecular structure maps. RDKit is an open source cheminformatics tool that enables the manipulation of compounds’ 2D and 3D molecular structures. It uses machine learning algorithms to generate compound descriptors and fingerprints, calculate compound structural similarities, and facilitate the visualization of 2D and 3D molecules, among other functions.

During the construction of the kinase molecular structure datasheet, the following steps are executed. Firstly, the SMILES for kinase data is obtained by connecting to the MySQL database (DuBois, 2014). Then, an SQL query is executed to extract the SMILES of the kinase data from the database. The query result is stored in a Python3.7 (Lutz, 2013) data structure. The RDKit library is then employed to transform the SMILES string into the chemical molecule structure diagram. By looping through the SMILES data, the Chem. MolFromSmiles () function is utilized to convert each SMILES into its corresponding molecular structure. Lastly, the resulting molecular structure diagrams are stored in binary format in the database. Due to MACCS keys (Taylor, 2024) simplicity and computational efficiency. The 166-bit structure of MACCS keys provides a sufficient level of detail for initial analyses, making them well-suited for broad feature-based screening and rapid processing of large datasets. While Extended Connectivity Fingerprints (ECFP) (Rogers and Hahn, 2010) or the Multilevel Atom Pair fingerprint (MAP4) (Capecchi et al., 2020) offer a higher resolution by capturing more detailed molecular structures and could potentially yield more nuanced insights, their complexity and computational demand are higher. Therefore, we have decided to stick with the simpler MACCS keys. MACCS keys fingerprints and the molecular descriptors are calculated and used to build models for predicting the logP (Partition Coefficient) oil-water partitioning (Wildman and Crippen, 1999). The molecular structure data for a particular kinase chemical is shown in Table 2.

**TABLE 2:** Chemical structure of kinase ligands: Representative examples.

Number	Name	Description
1	Smiles	COc1ccc (CNC(=O)c2ccc (-c3cc (-c4nnc(C)o4)ccc3C)cc2)cc1
2	Molecular Structure Diagram	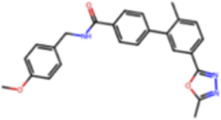
3	Highlight specific parts (oxygen atoms)	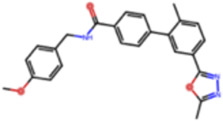
4	logP	logP (calculated by RDKit): 4.959040000000004 logP (calculated by Crippen): 4.959040000000004
5	MACCS keys fingerprint (166 bits)	00000000000000000000000000000000000000000000000000001000010000100100000000000000101100000000111110001000000000100100010011001111000000000100011111100111111111111111110

### 2.3 Platform construction and functional implementation

**FIGURE 4:**
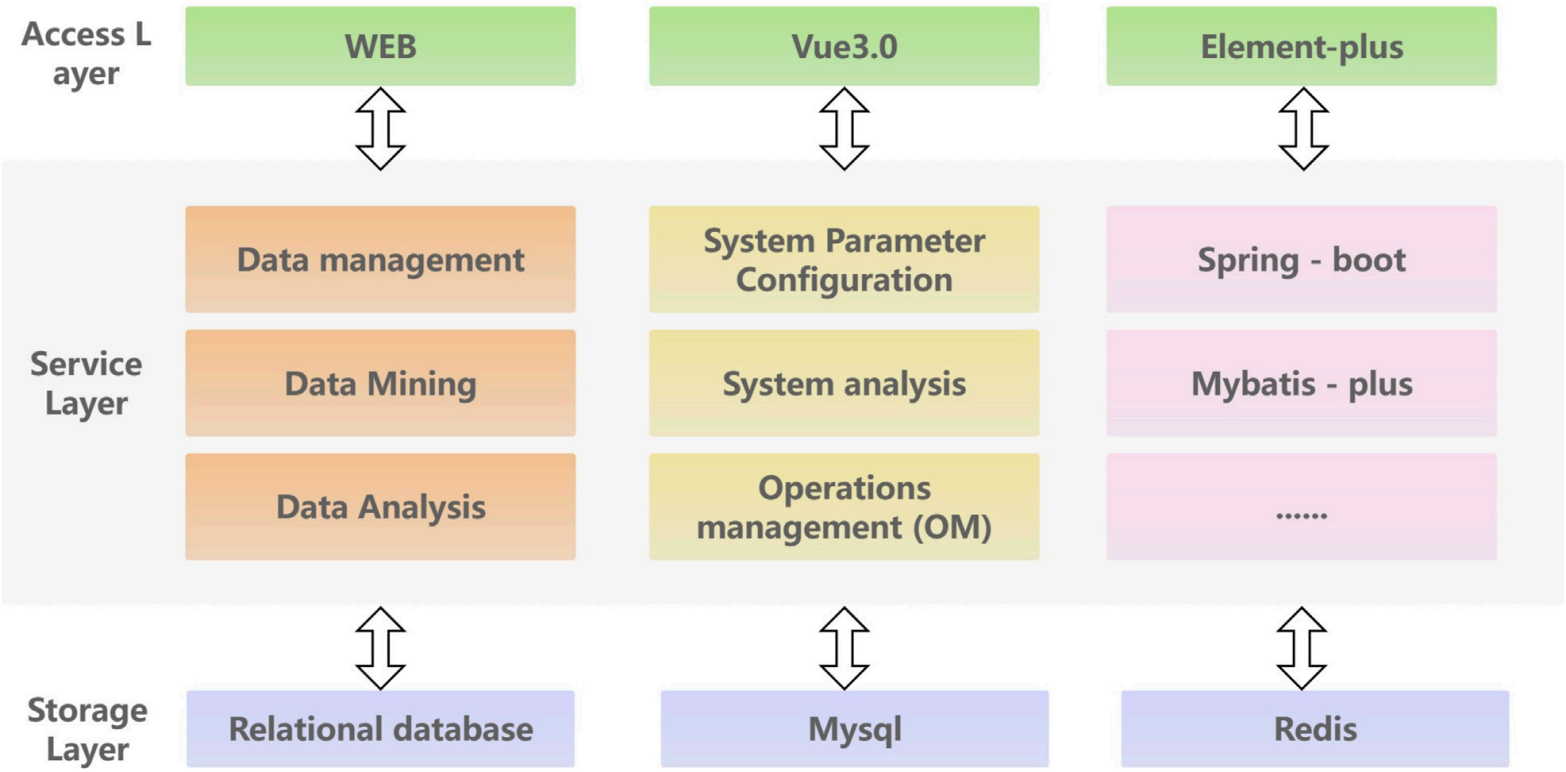
Detailed Architecture of the KLSD Database Platform. It describes a comprehensive architectural diagram of the KLSD database, highlighting key technologies and components.

To efficiently store, analyze and access kinase data, this paper proposes a database service framework based on spring Boot architecture. The front-end utilizes the modern Vue3 JavaScript framework to create a dynamic and high-performance user interface. The back-end utilized the spring Boot rapid development framework to build an efficient, reliable, and easily scalable back-end service. The MySQL relational database is chosen for data storage and management, with Redis caching utilized to improve system performance. The overall architecture follows a layered approach, comprising of an access layer, service layer and storage layer. The access layer primarily handles service invocation and user interaction, focus on tasks such as kinase data querying and kinase target calculation. The service layer is responsible for platform data management, intelligent analysis, and open queries to ensure the stable and effective operation of the service. The storage layer is responsible for data storage, ensuring high reliability and efficiency. Additionally, an external layer is provided to facilitate access to the data access service. The detailed platform architecture information of KLSD is shown in Figure 4.

The Target difference analysis of kinase activity is the core function of KLSD. To compare the biological activity of different compounds or molecules in kinase target analysis, pAct values provide a convenient way to do so. pAct values standardize the representation of activity intensity, making comparisons between different activity values easier. This simplifies the interpretation of the data and enables researchers to have a clearer understanding of the relative activity of different molecules or compounds. The pAct value compares activity strengths quantitatively. Generally, higher pAct values indicate stronger biological activity, while lower pAct values may indicate weaker activity. This can be used in assessing the relative potency of kinase inhibitors. Overall, the pAct value is an important metric in kinase target analysis. It simplifies the interpretation and comparison of data and provides strong support for drug design and optimization. pAct values are given in the following general formulae:

$$pAct=9-\mathrm{lg}\left(Act\right)$$

In the KLSD target difference analysis function, the user only needs to input two different targets and pAct value intervals. The platform will automatically retrieve all the relevant data for the two targets from the background and conduct the difference analysis. It will then provide the difference value between the two targets and all the relevant information for the corresponding compounds.

**FIGURE 5:**
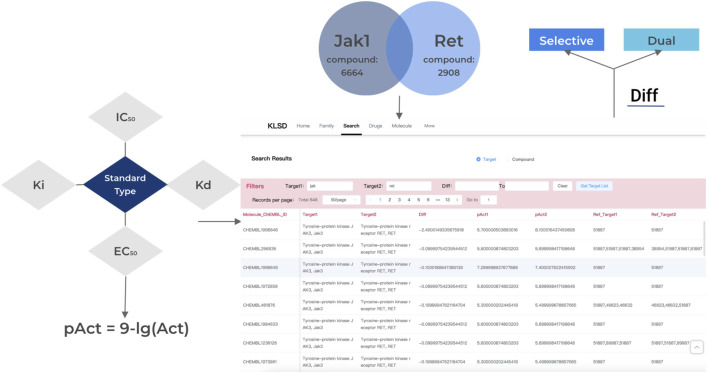
Comparative Differential Analysis of Kinase Targets. It describes the analytical methods used to compare kinase targets, highlighting aspects of variation and the conditions under which the analyses are performed.

To accomplish this function, the platform will first check the quality of the target-related information by eliminating abnormal values, missing data, or incorrect information. Only compounds with bioactivity_type corresponding to (p)Ki, (p) IC_50_, (p)Kb, (p)Kd or (p)EC_50_ (represented here as Act) were kept while those with activity_comments like “not active”, “inactive” were classified into an inactive group, and give an Act value of zero. In this study, our main focus was on analyzing SMKIs among all reported kinase targets. Therefore, we took the initiative to filter the Standard Type of compound activities. In our work, only (p)Ki, (p) IC_50_, (p)Kb, (p)Kd or (p)EC_50_ were included. Hence, we treat other types as abnormal value. It will then standardize the activity data, specifically those with measurement units of (p)Ki, (p) IC_50_, (p)Kb, (p)Kd or (p)EC_50_. These data will be converted into uniform concentration units, such as nanomolar (nM), to calculate the difference value between the targets. The platform will also determine the threshold for kinase activity. For example, compounds with an (p) IC_50_ below 10 nM (López-López et al., 2022) are generally considered to have high activity. The activity data will be categorized into two groups: active and inactive, based on this threshold. The platform will then analyze the correlation between the structural or characteristic differences of the compounds and their activity. Figure 5 displays the homepage of the database platform, the list of kinase data, and the target difference analysis.

KLSD is specifically designed to provide a comprehensive analysis of SMKIs across all reported kinase targets, setting it apart from broader databases. KLSD used data from the ChEMBL database, it has been observed that ChEMBL regularly updates its database, with a frequency of 1-2 updates per year. To ensure synchronization and real-time information, regular monitoring is conducted. Through the development of scripts that can automatically check for updates and download new data from ChEMBL, documentation is updated promptly to reflect changes in the data source. Our web platform has been designed with user accessibility in mind, featuring a user-friendly interface and advanced data visualization tools. These features make it easier for researchers, even those without extensive backgrounds in bioinformatics, to navigate the platform and extract valuable insights effectively.

## 3 Results and discussion

### 3.1 Statistics and analysis of data

Based on the actual research and the drug development process of kinases, we obtained a total of 138 families of SMKIs, Active and Inactive by analysing the most researched kinase families. Table 3 shows the top 15 kinase families in terms of the number of compounds.

**TABLE 3:** Detailed statistics of kinase family compounds.

Number	Family	Compound	Active	Inactive	Active (%)	Inactive (%)
1	NKF2	89762	4779	84983	5	95
2	MAPK	38070	21149	16921	56	44
3	Trbl	31060	7220	23840	23	77
4	PLK	27267	3214	24053	12	88
5	PKC	22872	14743	8129	64	36
6	CAMKL	19042	7103	11939	37	63
7	TBCK	18900	14757	4143	78	22
8	CDK	18620	15080	3540	81	19
9	PSK	16480	13061	3419	79	21
10	VEGFR	15303	13012	2291	85	15
11	EGFR	14112	10783	3329	76	24
12	Abl	14010	8666	5344	62	38
13	RSKR	12334	11458	876	93	7
14	PDGFR	10283	8648	1635	84	16
15	Jak2	9846	8637	1209	88	12

**FIGURE 6:**
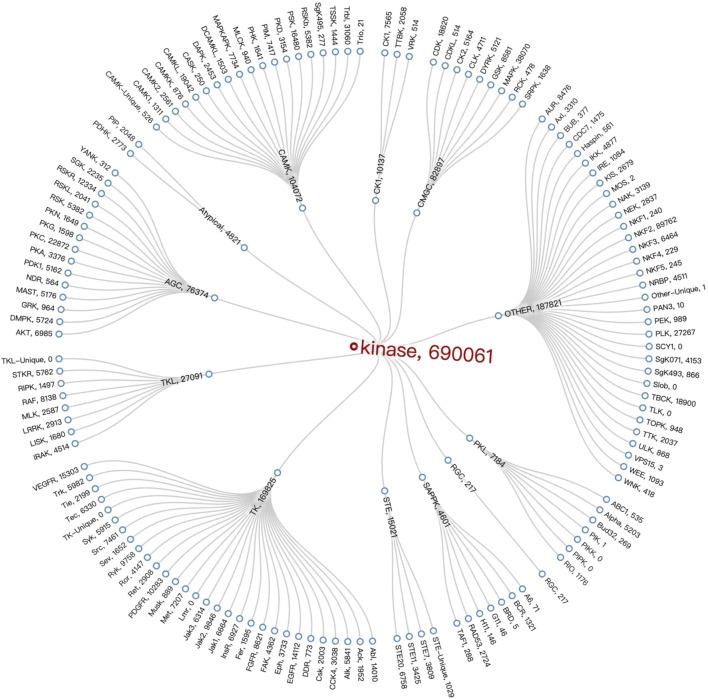
Kinase Family Radial Tree diagram. It employs specific technologies (Vue 3 and D3.js) that embody the interactive and data-driven nature of visualisation to represent the structure and number of compounds within the kinase family.

After analyzing the data on the compounds of kinase families, we employed Vue 3 and D3. js (Bostock et al., 2011) to create a radial tree diagram for visualizing kinase families data. Vue 3, a modern JavaScript framework, offers a reactive and composable architecture, making it ideal for developing interactive web applications. D3.js complements this by providing robust data-driven techniques for creating and manipulating graphical elements, which we used to calculate and render the complex structures of our radial tree diagram. It was observed that the TK Group had the highest number of compounds at 169,825. When looking at specific Families, the NKF2 Family, belonging to the OTHER Group, stood out with a number of compounds at 89,762, followed by the MAPK Family in the OTHER Group, which had a number of compounds at 38,070. The Radial Tree diagram illustrating the relationships between kinase families is displayed in Figure 6.

**FIGURE 7:**
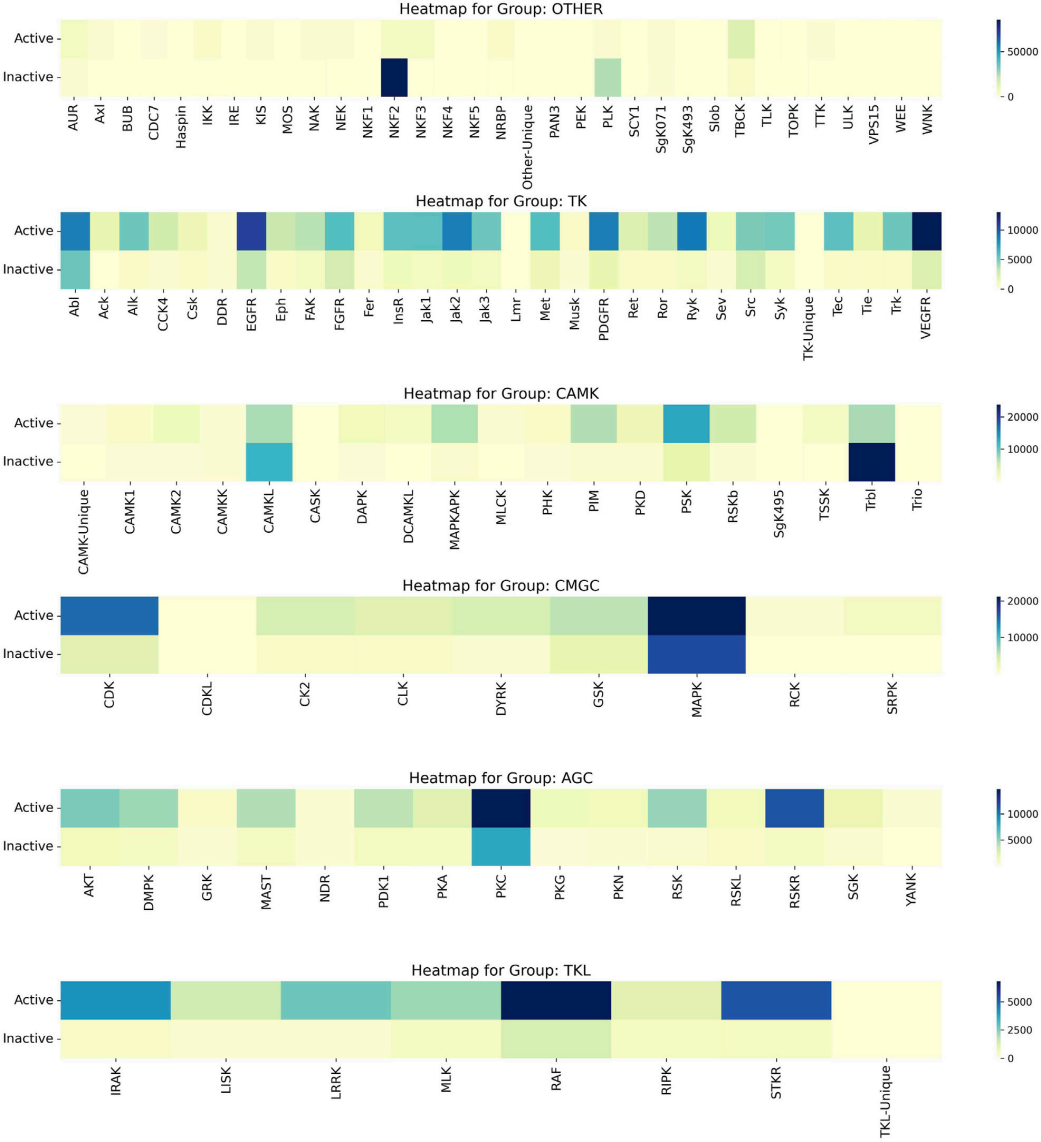
Heatmap of Kinase Families Activity. It uses heat maps to visually analyse the activity of the first 6 kinase families, reflecting the number of active and inactive. The varying color gradients in the heatmap signify different ranges in the number of compounds, with darker shades indicating higher numbers and lighter shades indicating lower numbers. Each color on the heatmap corresponds to specific range of compound numbers, as indicated on the right side.

Upon a thorough analysis of the activity profiles of compounds across different kinase families, we observed that the MAPK family within the CMGC group demonstrated the most significant number of active compounds. Specifically, while the MAPK family encompasses a total of 38,070 compounds, the number of active compounds identified was 21,149. This was followed by the CDK Family in the CMGC Group with an activity of 15,080. Furthermore, it is worth noting that the NKF2 Family had the highest inactivity of 84,983, and this was followed by the PLK Family in the OTHER Group with an inactivity value of 24,053. The heat map illustrating the activity of kinase families, known as Compound_Activity, is displayed in Figure 7. Figure 7 serves as an overall heat map that showcases the top six kinase families based on the number of compounds linked to each family.

The term “Standard Type” pertains to the specific metric employed to quantify the activity of compounds within the scope of pharmacological data. Common measurements under this category include (p)IC_50_, (p)EC_50,_ (p)Kd, and (p)Ki, among others. These parameters are pivotal as they denote the potency of a compound in its interaction with a biological target, be it through inhibition or activation. Understanding these measurements is fundamental to grasping the pharmacodynamics of the compounds under discussion. Furthermore, the ‘BAO label’, which stands for BioAssay Ontology label, provides a descriptor for the biological or pharmacological context within which an assay is conducted. This label encompasses classifications that can specify the nature of the target, such as a single protein, a protein complex, or a subcellular structure. The BAO label is indispensable for categorizing assays according to their biological specificity, thereby facilitating a clear interpretation of the experimental design and the resultant data.

**FIGURE 8:**
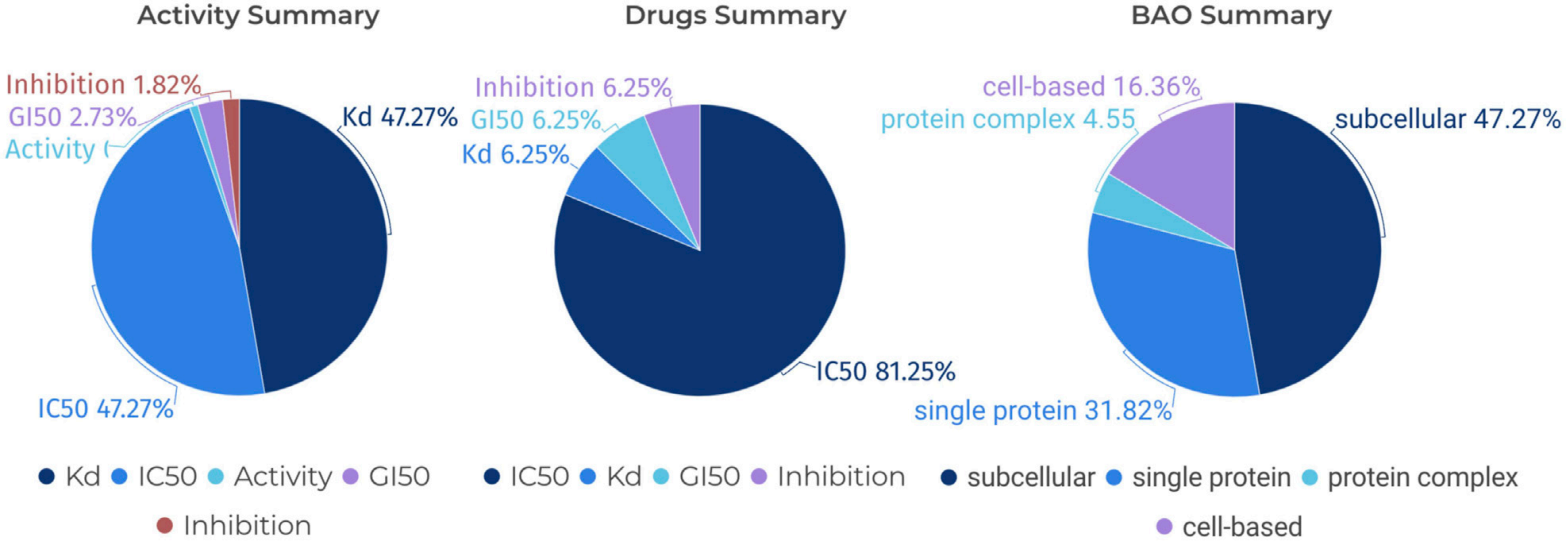
Pie Chart Analysis of Various Assays for a Specific Compound. It analyses a specific compound (CHEMBL3301607) in detail from three different perspectives: Standard Type for Activity, Standard Type for Drugs and BAO Label for BAO.

In the field of kinase research, the study of JAK1 (Janus Kinase 1) is crucial as JAK1 plays a significant role in signaling pathways that impact cellular processes, including immune response and inflammation (Harris and Cummings, 2021). FILGOTINIB, a selective inhibitor of JAK1, is proven to be highly effective in the treatment of rheumatoid arthritis (Feagan et al., 2021). Therefore, we have used CHEMBL3301607, which corresponds to Filgotinib, as an example in Figure 8. Specifically, we can search for “Filgotinib” in the “Drugs” section of the KLSD database, the platform automatically retrieves the corresponding compound name, Standard Type, and BAO Label among other information. These pieces of information are crucial for understanding the mechanisms of action of drugs, evaluating drug efficacy, and optimizing drug design. As shown in Figure 8, we analysed CHEMBL3301607 from three perspectives: the standard type, the standard type of the drug and the BAO label of the BAO. This approach not only enhances our understanding of Filgotinib’s role as a JAK1 inhibitor but also demonstrates the KLSD database’s utility in facilitating detailed drug analysis and research involving SMKIs.

### 3.2 Conclusion

Currently, the kinase database platform has accumulated a substantial amount of kinase-related data, including information on kinase structure, function, regulation, and biological roles. KLSD can provide personalized data query, analysis, and display tailored to researchers’ specific needs. By analyzing the kinase data, KLSD seeks to identify patterns and features, offering valuable insights for research and improving researchers’ efficiency. Furthermore, KLSD ensures data quality and accuracy, making it highly beneficial in fields such as biomedical research and drug discovery. However, there is still ample room for growth in kinase deep learning and artificial intelligence. We must leverage artificial intelligence technology to further analyze large-scale kinase data, uncover potential associations and patterns, predict kinase functions and biological effects, and broaden the functionality and application areas of the kinase database platform. These advancements will enable users to conduct more comprehensive studies and analyses beyond the current capabilities of the platform, meeting the evolving needs of users and providing greater support and resources for kinase research.

## Data Availability

The original contributions presented in the study are included in the article/supplementary material, further inquiries can be directed to the corresponding authors.
